# Urban Health and Social Marginality: Perceived Health Status and Interaction with Healthcare Professionals of a Hard-to-Reach Community Living in a Suburban Area of Rome (Italy)

**DOI:** 10.3390/ijerph18168804

**Published:** 2021-08-20

**Authors:** Susanna Caminada, Federica Turatto, Silvia Iorio, Lorenzo Paglione, Miriam Errigo, Elena Mazzalai, Anissa Jaljaa, Dara Giannini, Marco Tofani, Maria Benedetta Michelazzo, Adelaide Landi, Massimo Napoli, Maria Alessandra Brandimarte, Livia Maria Salvatori, Aurora Angelozzi, Giovanni Baglio, Enrico Di Rosa, Alessandra Battisti, Maurizio Marceca

**Affiliations:** 1 Department of Public Health and Infectious Diseases, Sapienza University of Rome, 00185 Rome, Italy; federica.turatto@uniroma1.it (F.T.); elena.mazzalai@uniroma1.it (E.M.); anissa.jaljaa@uniroma1.it (A.J.); dara.giannini@uniroma1.it (D.G.); marco.tofani@uniroma1.it (M.T.); aurora.angelozzi@uniroma1.it (A.A.); maurizio.marceca@uniroma1.it (M.M.); 2 Department of Medico-Surgical Sciences and Biotechnologies, Sapienza University of Rome, 00185 Rome, Italy; silvia.iorio@uniroma1.it; 3 Department of Civil, Constructional and Environmental Engineering, Sapienza University of Rome, 00184 Rome, Italy; lorenzo.paglione@uniroma1.it; 4 Department of Prevention, Local Health Unit Roma 1, 00135 Rome, Italy; adelaide.landi@aslroma1.it (A.L.); massimo.napoli@aslroma1.it (M.N.); ale.brandimarte@aslroma1.it (M.A.B.); enrico.dirosa@aslroma1.it (E.D.R.); 5 Department of Social Sciences and Economics, Sapienza University of Rome, 00185 Rome, Italy; errigo.1476440@studenti.uniroma1.it; 6 Section of Hygiene, Institute of Public Health, Università Cattolica del Sacro Cuore, 00168 Rome, Italy; mbenedetta.michelazzo@gmail.com; 7 District 3, Local Health Unit Roma 1, 00139 Rome, Italy; livia.salvatori@aslroma1.it; 8 Research and International Relations Office, Agenzia Nazionale per i Servizi Sanitari Regionali (AGENAS), 00187 Rome, Italy; baglio.giovanni@yahoo.it; 9 Department of Planning, Design, and Technology of Architecture, Sapienza University of Rome, 00196 Rome, Italy; alessandra.battisti@uniroma1.it

**Keywords:** urban health, social determinants of health, health inequities, hard-to-reach groups, health-related behaviours, healthcare use

## Abstract

The study reports an urban health investigation conducted in Bastogi, an outskirt of Rome (Italy) characterised by social marginalization and deprivation. Our aim was to analyse the health perception, health-related behaviours, and interaction with healthcare professionals of the inhabitants of Bastogi compared to the population living in the area of the same local health unit (ASL). The Progresses of Health Authorities for Health in Italy questionnaire (PASSI) was administered to a sample of 210 inhabitants of Bastogi. Data were analysed and compared to those of the ASL collected in 2017–2018. The socio-economic indicators showed an overall worse condition for the inhabitants of Bastogi, with a significantly higher proportion of foreign and unemployed residents and a lower educational level compared to the ASL. Significant differences in the prevalence of non-communicable diseases, mental health complaints, and participation in prevention strategies, including cancer screening, were found. The questionnaire showed a lower help-seeking behaviour and a lack of reliance on health professionals in Bastogi inhabitants. Our findings highlight how social determinants produce health inequities and barriers to accessing healthcare. The difficulties of conducting quantitative research in complex and hard-to-reach contexts, characterized by high social vulnerability, are outlined.

## 1. Introduction

Living conditions are a well-acknowledged determinant of health and health inequalities [1,2,3]. As urban living becomes the predominant social context for most of the world’s population, it is increasingly urgent to investigate the broad physical, social, and economic determinants that influence the health of city dwellers [4,5,6]. In particular, communities experiencing residential segregation and social marginalization have disproportionate exposure and susceptibility to a combination of social determinants and risk factors associated with worse health outcomes [6,7,8].

The issue of urban health and marginalised communities has gained increasing attention in the last decade in Italy as well [9,10]. Several studies have been conducted in different urban areas with the aim of identifying specific mechanisms and solutions [11,12]. In this context, a multidisciplinary, action-research project based on the theoretical foundations of health promotion [13,14] and on the community-based approach [15] was set up to create a research and intervention model capable of adequately capturing the complexity of the relation between socio-environmental distress and health inequalities from an ecological perspective of health. We incorporated different approaches deriving from sociology, architecture/urbanism, medicine/public health, history, and bioethics by means of an institutional collaboration involving the corresponding departments of Sapienza University of Rome and, through an inter-institutional collaboration, integrating the following local, regional, and national entities: the Local Health Unit (ASL) of the Regional Public Health System covering the area of Bastogi (ASL Roma 1), the Municipality XIII of Rome, and the Department of Epidemiology (DEP) of the Lazio Region. The general aim of the project was to build a qualitative-quantitative research infrastructure specifically adapted to the urban context of reference, advocating the institutional assumption of the socio-sanitary needs of the community—which was both the object and subject (in a logic of empowerment) of the study. 

The setting of the project is an area characterized by strong social vulnerability located in northwest Rome, known as the ex-Bastogi residence, named after the building contractor who built it (henceforth referred to as Bastogi). The residential nucleus of Bastogi is an enclave in the urban fabric, characterized by multi-story buildings set in a metropolitan connective tissue of little value and quality: a sort of island in the city, located on a hill and surrounded by perimeter walls, spatially segregated from the outside and poorly served by public services. In this area, the social degradation of groups living in serious socio-economic and occupational distress is combined with the degradation of buildings caused by the physical decay of housing structures and outdoor public spaces, which make the whole area distinctively marginalized [16]. A considerable part of the inhabitants is not formally registered, being illegal tenants often living in overcrowded conditions: data from the ISTAT’s (Italy’s National Statistics Institute) general Census of population and housing of 2011 showed that with approximately 2000 inhabitants estimated, only 1033 were listed. The effect of the centre-periphery social gradient in the distribution of determinants, such as employment, education, income, and building status, was confirmed by a recent study: significant differences were found between the two populations particularly with regards to education and building status [16]. A previous analysis concerning the access to healthcare services showed a higher use of hospital care (both emergency and inpatient) on the part of the population of Bastogi compared to the neighbouring areas and to the whole city of Rome [17]. The underlying factors that might explain this phenomenon and whether it can be attributed to the relationship with the local socio-health services of the inhabitants of Bastogi or to their perception of health are still unclear.

The aim of the study was to characterize the community health profile of the people in Bastogi in order to verify the existence of inequalities in perceived health and in the population’s relation with public health services compared to that of the reference population of the entire Local Health Unit of which Bastogi is part (ASL Roma 1) through the use of the PASSI survey (Progress of Health Authorities for Health in Italy) [18]. 

## 2. Materials and Methods

The PASSI survey is part of the Italian behavioural risk-factor surveillance system. It was developed in 2006 by the Italian Ministry of Health as an instrument to monitor the health status of the Italian population and the achievement of the goals set in the National and Regional Health Plans [18]. The PASSI survey is usually administered by phone to a representative sample randomly extracted from the list of enrolled residents in each Local Health Unit, aged from 18 to 69 years, stratified by sex and age groups based on the proportion of the local population in each of these strata [19]. Inclusion criteria include residence in the Local Health Unit area and having an available telephone number; exclusion criteria include inability to understand Italian, inability to take part in the interview, and being hospitalized or institutionalized. The questionnaire covers a variety of topics for health-related behaviours and prevention, including topics related to individual health status, lifestyle, and offer and uptake of health prevention programs (including preventive cancer screening). Particular attention is given to subjective aspects, such as the respondents’ perceptions, opinions, knowledge, and attitudes about health behaviours, and whether their doctors provide them with appropriate medical advice. The questionnaire includes a standard set of 114 questions grouped into 15 modules. Almost all questions are closed-ended with multiple-choice answers. Many questions are administered only to specific population subgroups. All data are self-reported, and questionnaire data are collected anonymously [20].

For the purpose of this study, the research team collaborated with ASL Roma 1, the Local Health Unit covering over a million inhabitants of the central-northern part of Rome (36.3% of the total population of the city). The questionnaire was administered between March 2018 and June 2019 (using the format of the previous year’s questionnaire) to a sample of 210 people among the 642 census-registered residents of Bastogi who satisfied the inclusion criteria. A random sample of respondents, selected according to the stratification criteria applied in the national PASSI survey strategy, with eventual substitutions of the primary target with correspondent socio-biographical characteristics, was reached by phone. Due to the low response rate, door-to-door interviews were carried out to a convenience sample in order to reach the target sample size. Therefore, the survey was conducted partly by phone and partly face-to-face by trained personnel of ASL Roma 1 and Sapienza University.

### Analysis

We analysed the data of the sections related to (1) health perception; (2) health-related behaviours, including the uptake of prevention programs; and (3) the quality of interactions with health professionals. We compared the results of the Bastogi group with those of the entire ASL Roma 1 routinely collected in the years 2017–2018. Descriptive statistics of the items included in the questionnaire were performed. Continuous variables are expressed as means (standard deviation SD). Categorical variables are expressed as proportions. Univariate analysis was performed using chi-square test and Fisher’s exact test—where appropriate—for categorical variables and Student’s *t*-test for continuous variables. *p*-values ≤ 0.05 were considered statistically significant. All analyses were performed by a subgroup of the research team with STATA 13 (StataCorp LLC, 4905 Lakeway Drive College Station, TX, USA).

## 3. Results

### 3.1. Socio-Demographic Characteristics

The mean age of the respondents in the Bastogi group was 44.1 (SD 1.0) years, which was not significantly different from the mean age in the ASL Roma1 group: 45.4 (SD 0.5) years. Socio-demographic characteristics of the respondents are summarized in Table 1. Among the most interesting results, we observed that 64.9% of the respondents in the Bastogi group interrupted their schooling after middle school compared to 14.9% in ASL Roma 1; there was a significantly higher proportion of foreign residents in Bastogi compared to ASL Roma 1 (*p* = 0.002); only 40.2% of the Bastogi sample stated that they were continuously employed at the moment of the interview; and 73.1% of the respondents stated that they had considerable or some difficulties in coping with normal monthly expenses compared to 40.8% in the ASL Roma 1 group (*p* < 0.001).

### 3.2. Health Status: Self-Reported Perception of Health and Health-Related Behaviours

The percentage of respondents in the Bastogi group who stated that, in general, their health was “good” or “very good” was 58.1%, while 15.2% answered “bad” or “very bad,” compared to 71.6% and 4.7%, respectively, in the ASL Roma 1 group (Table 2). On average, respondents from Bastogi claimed they had not felt physically well for 6.5 (SD 10.4) days over the previous 30 days compared to 1.7 (SD 4.3) among respondents of ASL Roma 1 (*p* < 0.001). When considering psychological distress, the Bastogi group reported not feeling well for an average of 8.3 (SD 11.4) days over the previous 30 days compared to 2.3 (SD 1.9) days in the ASL Roma 1 group (*p* < 0.001). A substantial proportion of respondents from Bastogi (56.2%) were current smokers (ASL Roma 1: 25.8%; *p* < 0.001), while only 26.2% stated that they had never smoked (ASL Roma 1: 53.7%; *p* < 0.001). A total of 56.7% reported they had practiced moderate/intense physical activity in the previous 30 days (ASL Roma 1: 61.3%; *p* = 0.271), but only 16.2% reported intense physical activity (ASL Roma 1: 24.9%; *p* = 0.007). Among the respondents from Bastogi, 42.9% considered their body weight to be too high, 6.2% too low, and 51.0% appropriate (compared to 32.7%, 4% and 61.9%, respectively, in ASL Roma 1, *p* = 0.008 *). Based on self-reported body weight and height, the mean value for body mass index (BMI) [21] among the Bastogi group was 26.3 (SD 0.8) kg/m^2^, while among the ASL Roma 1 group, it was 23.6 (SD 0.1), *p* < 0.001. The prevalence of chronic diseases reported by the respondents is summarized in Table 2. The proportion of respondents who reported they had been diagnosed with diabetes, obstructive pulmonary diseases, and acute and chronic cardiovascular diseases was significantly different between the two samples.

### 3.3. Interaction with Healthcare Professionals

Table 3 summarizes the results related to the interaction with health professionals. When not otherwise specified, when N is different from the sample size, missing answers are due to non-respondents.

#### 3.3.1. Health-Related Behaviours

The respondents in the Bastogi group stated that in the last 12 months, their doctor had inquired about their smoking habits (51.4%) but had not asked them about their weight (51.2%) or their drinking habits (60.6%). These results were significantly different from those in ASL Roma 1 shown in Table 3. Among current smokers, the majority in the Bastogi group (55.4%) stated that they had not received any advice on quitting from their doctor (vs ASL Roma 1: 36.2%, *p* < 0.001).

#### 3.3.2. Cardiovascular Risk Assessment

While most of the respondents from both groups stated they had their blood pressure measured at least once by a doctor, 7.1% of the respondents from Bastogi aged 35–69 years stated that a doctor had calculated their cardiovascular risk compared to 0.6% in ASL Roma 1 (*p* < 0.001).

#### 3.3.3. Immunisation

While there was no statistically significant difference in the uptake of the flu vaccine (Table 3), the proportion of women aged 18–49 years from Bastogi who reported being vaccinated against rubella was higher than in ASL Roma 1 (54.7% and 34.6%, respectively, *p* = 0.005).

#### 3.3.4. Help-Seeking Behaviour

Help-seeking behaviour was investigated only in respondents who stated they had experienced mental distress for more than 7 days. Among these respondents, 49 were from ASL Roma 1 (5.5% of the sample) and 69 from Bastogi (32.9%). A total of 49.3% of the respondents in the Bastogi subgroup stated they did not contact anyone when experiencing mental distress, although the results were not significantly different from the comparison group (34.7%). Healthcare professionals were contacted by 29% of respondent from Bastogi and 44.9% from ASL Roma 1 (*p* = 0.075), while support from friends and family was sought by 28.6% of Bastogi respondents (ASL Roma 1: 12.2%, *p* = 0.034).

### 3.4. Screening Tests

The questions concerning Pap smear were only addressed to women aged 25–64. As reported in Table 4, 90.8% of the respondents from Bastogi claimed to have taken a Pap smear at least once in their life, with no significant difference with respondents from ASL Roma 1 (94.3%). Among the Bastogi group, 17.2% had undergone a Pap smear more than 5 years before, 11.1% 3–5 years before, 30.3% 1–3 years before, and 41.4% in the last 12 months (N = 99). These results were significantly different from those of the ASL Roma 1 group (*p* < 0.001), in which 3.2% had undergone a Pap smear more than 5 years before, 6.4% 3–5 years before, 35% 1–3 years before, and 55.4% had undergone the test in the last year (N = 377). A total of 67% of the respondents from Bastogi reported that they had received a letter from the local health unit inviting them to take the Pap smear compared to 47.2% in ASL Roma 1 (*p* < 0.001). A total of 72.8% of women from ASL Roma 1 reported that a doctor or healthcare professional had advised them to undergo the Pap smear regularly, even in the absence of symptoms, while only 55.8% of respondents from Bastogi claimed to have received adequate counselling (*p* < 0.001). However, analysing the reasons why women from Bastogi reported not having taken a Pap test recently, only 6.25% reported not having received counselling, while 31.3% admitted the reason was laziness, 12.5% reported fear, and 12.5% lack of time. Similarly, among the reasons given by women from ASL Roma 1 for not taking the test recently, the most frequent answers were laziness (30.4%) and lack of time (16.1%), followed by the perception of not needing it (12.5%), while only 5.4% claimed they were not advised to take it. A total of 74.3% of the respondents from Bastogi who had undergone a Pap smear reported they were tested for free, while 10.8% paid a co-pay fee [22], and 12.2% paid out of their own pocket. This was significatively different from the respondents from ASL Roma 1: 51.6% paid out of their own pocket the price of the test (*p* < 0.001).

The questions concerning breast cancer screening were only addressed to women aged 40–69. As reported in Table 4, 75.3% of respondents from Bastogi claimed they had got a mammogram at least once in life compared to 90.4% in ASL Roma 1 (*p* = 0.001). A total of 23.1% of the Bastogi respondents reported undergoing the test more than 2 years before, 30.8% 1–2 years before, 44.6% in the last 12 months, while 1.5% did not remember. No significant differences were found compared to the ASL Roma 1 group (N Bastogi = 65, N ASL = 300). Among respondents from Bastogi, 21% reported that they had never received the letter from the Local Health Unit inviting them to get a mammogram against the 12.8% in ASL Roma 1 (*p* = 0.005). A total of 71.7% of the women from ASL Roma 1 reported that a doctor or healthcare professional had advised them to get a mammogram regularly compared to 78.3% in the Bastogi group. The main reasons why women from Bastogi reported not getting a mammogram recently were laziness (30%), pain/discomfort (20%), and thinking it was not necessary (20%). Among the reasons for not taking the test recently given by women from ASL Roma 1, with no significant difference from Bastogi respondents, the most frequent were embarrassment (35%), laziness (32.5%), and pain/discomfort (7.5%). 

A total of 82.7% of the respondents from Bastogi who reported getting a mammogram did not incur any expenditure, while 9.6% paid just a co-pay fee, and 7.7% paid out of their own pocket. This was significatively different from the respondents of ASL Roma 1, among whom 36.1% paid for a private test (*p* < 0.001).

## 4. Discussion

The mean age of the respondents from Bastogi was not significantly different from the one of ASL Roma 1 respondents. However, the difference in the gender composition of the two samples was significant, with the Bastogi group being composed to a greater extent by women, and the ASL Roma 1 group reporting an even share of male and female interviewees. These differences could be explained by the sampling strategy adopted and by the fact that, as shown in previous studies, in the Bastogi community, women are usually more available to collaborate and also because men are busier with their jobs or subject to legal/criminal restrictions [23]. The indicators of socio-economic status (SES) showed an overall worse condition among the inhabitants of Bastogi. The educational level was lower than the one reported by the ASL Roma 1 respondents, and the share of unemployed and non-continuously employed people among the Bastogi group was significantly higher, being more likely to demonstrate perceived financial and job insecurity. These factors are acknowledged determinants of health inequalities and related to socioeconomic disadvantages [24,25,26]. In particular, a lower educational attainment is a strong predictor of a low health literacy [27]. In such contexts of urban social marginalization, the inhabitants present a higher level of social fragility and vulnerability [6,28,29]. In Italy, due to the poor legislation that does not sufficiently promote and support the integration of immigrants, a greater presence of foreigners can be found in this specific type of milieu, characterized by high social vulnerability [30]. Indeed, in Bastogi, we registered more than double the share of foreign population in comparison to the ASL Roma 1 sample.

There was a significant difference in the self-rated health between the inhabitants of Bastogi and the population of ASL Roma 1, with the first group reporting three-fold higher perception of “bad”/“very bad” health. Self-rated health, a commonly used subjective measure of overall health status, is an important indicator of quality of life that can be strongly conditioned by the characteristics of the neighbourhood [31,32]. Bastogi is a deprived and disadvantaged area marked by poverty and social exclusions. These factors all contribute to influencing the level of social capital [33]. It has been shown that, in deprived sub-groups, social capital is more strongly associated with common mental disorders, such as major depression and substance abuse [34,35], and to a lower self-reported health status [36]. In Bastogi, the high proportion of self-reported diagnoses of chronic conditions such as diabetes (6.7%), obstructive pulmonary diseases (19.5%), and cardiovascular diseases (10.4%) is not only higher than in ASL Roma 1 but also higher than the national data from PASSI 2016–2019 surveillance, which is, respectively, 4.7%, 6.8%, and 4.6% for cardiovascular diseases [37]. These data confirm that social marginalization significantly shapes the risk of chronic degenerative diseases, as shown by a growing body of literature [38,39,40]. In fact, in Bastogi this could also be due to the poor housing conditions and low indoor environment quality of this urban area described in a recently published study [41]. Investigating the risk factors connected with these diseases, the group from Bastogi reported on average a higher BMI, practiced less intense physical activity, and consisted of a much higher proportion of smokers. The latter figure of prevalence of current smokers in Bastogi (56.2%) is extremely relevant when compared to the national (25.3%) and regional (27.9%) percentages extracted from PASSI 2016–2019 [37]. These data could explain the prevalence of chronic diseases, as an unhealthy lifestyle is related to non-communicable diseases [42]. Unhealthy behaviours are more frequent among the population reporting a lower SES, as the one observed in the Bastogi context [43,44]. In general, a disadvantaged socio-economic condition is a recognized risk factor for non-communicable-diseases and for an overall worse health status [17,26]. 

The proportion of respondents who stated they had not had any contact with health professionals in the last year was higher in the Bastogi group compared to the ASL Roma 1 group in all the items of the questionnaire. The lack of reliance on health professionals was especially noticeable when asked about help-seeking behaviours when experiencing mental distress. This is consistent with previous evidence on the inhabitants of this area. In fact, both qualitative and quantitative studies have shown that the Bastogi community is a hard-to-reach population, experiencing barriers to access and inappropriate use of health services. Potential factors contributing to this situation may include geographical barriers, social isolation, lack of trust towards institutions, and the perception of being neglected by the public institutions [45,46]. However, our study pointed out that among respondents who had had contact with a health professional, the proportion of those reporting a perceived interest about their health-related behaviours was higher than in ASL Roma 1. This difference was particularly marked in questions concerning cardiovascular disease risk factors, especially smoking and alcohol habits and healthy weight. These results are compatible with those that emerged from an assessment of the possible stigma of public primary care professionals towards the inhabitants of Bastogi [45]. These results highlight how most likely the staff of public primary care services are sufficiently sensitive and competent, but above all, they have the required level of knowledge of the contexts in which they operate to understand the need for greater attention regarding social determinants of health. These results suggest a different situation compared to other studies on the same issue [47,48]. Further studies are needed to investigate which combination of factors leads to these relatively higher levels of awareness regarding increased vulnerability amongst health professionals.

The results of the items concerning the uptake of cancer screening programs suggest a lower participation among the Bastogi respondents. These findings are consistent with the scientific literature on the subject, which identifies socioeconomic status as an important determinant contributing to screening uptake [49,50]. Similar results were found in other studies conducted in Italy as well [51,52]. Among women who had undergone a Pap smear or a mammogram, the proportion of those who did not pay for the test was higher for the Bastogi group compared to ASL Roma 1. In Italy, the Regional Health Systems actively provide screening tests free of charge for the population included in the program (women aged 50–69 for breast cancer screening every 2 years, women aged 25–64 for cervical cancer screening every 3 years). Our results therefore suggest that women from Bastogi relied mostly on the Public Health System to perform the test, while women in ASL Roma 1 turned more to the private sector. This could be explained by the higher proportion of women of Bastogi who claimed to have received the invitation letter, although it should be considered that the women who turned to the private sector or performed the test spontaneously may not have paid attention to the ASL communication.

### Study Limitations

Our study has some limitations. First, in order to cope with the difficulties of recruiting respondents in a hard-to-reach population such as that of Bastogi, the sampling strategy for this group was adapted to the context. The sample was selected exclusively from the population registered in the ASL data register (based on the Census), which does not completely correspond to the entire population living in the Bastogi area. Therefore, the irregular and extremely marginalised portion of the population, which might probably present even greater socio-health fragility, was excluded. Accordingly, the differences recorded between the two populations surveyed may be underestimated. To overcome the challenge of reaching this “hidden population”, in the absence of administrative data, innovative approaches are required in future investigations [53,54]. Moreover, due to the constraints and the barriers induced by this specific context, part of the interviews was conducted face-to-face, unlike the strategy adopted at a national level and in ASL Roma 1 for the same survey. This strategy enabled a better engagement with the community and improved its participation [55]. Although conducting in-person interviews represents a variation of the validated method of administering the PASSI questionnaire, the “PASSI D’Argento” (literally “Silver PASSI”, a similar survey conducted specifically on the population aged >65 y) verified the reproducibility between the two methods [56]. However, we are aware that the different administration may have influenced the results of our study. First of all, this could explain the over-representation of female respondents, who had a higher probability of being reached by the interviewers on the field. Moreover, the PASSI Surveillance System is based on self-reported data and therefore possibly affected by desirability bias and recall bias, which could be affected by the mode of administration of the questionnaire [57]. In particular, the desirability bias may have been higher for face-to-face interviews compared to phone interviews [58,59]. Despite these limitations, we are convinced that—for the relevant purposes of the public health approach—some methodological adjustments were necessary to be able to investigate the health inequities of this specific community. Finally, differences in educational attainment and health conditions could have affected the reliability of some answers [60]. The analysis of these data did not include a correlation with specific measures of health literacy, which, as suggested by educational levels, may have influenced the ability to fully understand some questions and thus the reliability of the corresponding answers. The inclusion of a health literacy variable could help understand the influence of this factor on health-related behaviours and therefore should be included in future studies. 

Conversely, the most important strengths of this study were the direct involvement of the local health authority in all steps of the research and the achievement of having quantified and given evidence of an important health gap within the same territory, of which the ASL must be aware to tackle it through the necessary actions.

## 5. Conclusions

Our research confirms the importance of investigating the health of groups and communities characterized by social marginality and deprived living conditions, such as that of Bastogi area in Rome. The study has shown how it is possible to conduct quantitative research and develop comparative analysis of exposure to risk factors or preventive interventions even in complex contexts characterized by high social vulnerability and normally escaping the institutional systems of monitoring and surveillance. In particular, the comparison between the population studied and the wider population living in less deprived neighbouring urban contexts can highlight the effect of the main social determinants in the production of inequities in health and/or healthcare. In our study, it was possible to demonstrate a relevant impact of the social gradient, which in turn was correlated with the specific characteristics of the urban area considered. The research has some limitations due to the complexity of the context, which required an adaptation of a nationally validated methodology. However, it was still capable of providing elements of analysis relevant to public health. We believe that this approach is necessary to reach more complex realities and, possibly, to generate tools that could be used also for other existing hard-to-reach realities both nationally and internationally [52].

The involvement of health and social public institutions can foster an increase in awareness with respect to these urban areas of distress and thus promote policies aimed at responding to the needs of these vulnerable inhabitants. The social sensitivity of physicians and other healthcare professionals plays a central role; our research seems to show a comforting attention of health personnel in identifying situations of social risk and directing healthier behaviours. In our context, the ASL participation in the monitoring activities has been accompanied by targeted actions aimed at encouraging the inclusion of the Bastogi population within the clinical pathways of the National Health System. In particular, the ASL Roma 1 has promoted the development of a “usability map”, providing useful information to the population about the use and access of social and health services. This activity culminated in the activation of a citizen help desk, named “Unified Access Point,” aimed at informing, orienting, and allowing direct access for the local community to most different services (certifications of disability, social and health care, multidimensional assessment activities, etc). Some interventions, such as a Health Promotion Day, have been carried out in collaboration with Sapienza University and with the territorial administration of the XIII Municipality of Rome and the NIHMP (National Institute for Health, Migration, and Poverty), with mobile clinics for the execution of screening tests, informative panels promoting good nutrition, and recreational aggregation events. 

Further studies are needed to evaluate the impact on the target population’s perceived health and relation with healthcare services after the implementation of such initiatives, although an integrated approach is necessary to tackle health inequity within the urban context [61].

## Figures and Tables

**Table 1:** Selected socio-demographic characteristics of the respondents. Results are expressed as absolute values and percentages.

	Bastogi		ASL		p
**Gender**	n	%	n	%	
Male	76	36.2	421	46.8	0.005
Female	134	63.8	478	53.2	
**Educational level**	n	%	n	%	
None	5	2.4	1	0.1	<0.001 *
Elementary school	19	9.1	12	1.3	
Middle school	111	53.4	121	13.5	
High school	60	28.9	431	47.9	
University degree	13	6.3	334	37.2	
**Marital Status**	n	%	n	%	
Single	97	46.4	345	38.4	<0.001
Married	62	29.7	467	52.0	
Separated/Divorced	35	16.8	68	7.6	
Widowed	15	7.2	19	2.1	
**Citizenship**	n	%	n	%	
Italian	185	88.9	851	94.7	0.002
Foreign	23	11.1	48	5.3	
**Employment status**	n	%	n	%	
Unemployed	94	45	257	28.6	<0.001
Continuously employed	84	40.2	593	66.0	
Non-continuously employed	31	14.8	49	5.5	

* Fisher’s exact test.

**Table 2:** Absolute and relative frequencies of responses to the PASSI questionnaire. Domain: Self-reported health status and prevalence of chronic diseases. Results are expressed as absolute values and percentages.

	Bastogi		ASL		*p*
	N = 210		N = 899		
** How would you describe your health?**	n	%	n	%	
Very good	27	12.9	100	11.2	<0.001
Good	95	45.2	544	60.5	
Not bad	56	26.7	213	23.7	
Bad	25	11.9	34	3.8	
Very bad	7	3.3	8	0.9	
** Have you ever been diagnosed with?**	n	%	n	%	
Diabetes	14	6.7	28	3.1	0.015
Kidney failure	2	1.0	10	1.1	1.000 *
Obstructive pulmonary diseases	41	19.5	53	5.9	<0.001
Cardiovascular diseases	22	10.4	32	3.6	<0.001
Cancer	10	4.8	46	5.1	0.833

* Fisher’s exact test.

**Table 3:** Absolute and relative frequencies of responses to the PASSI questionnaire. Domain: Interaction with healthcare professionals. Results are expressed as absolute values and percentages.

	Bastogi		ASL		*p*
	N = 210		N = 899		
** In the last 12 months, has a doctor ever**	n	%	n	%	
* asked if you smoke?*					
Yes	108	51.4	348	38.7	0.003 *
No, nobody asked	82	39.1	474	52.7	
No, I haven’t spoken with any health worker	19	9.0	72	8.0	
I do not know	1	0.5	5	0.6	
* advised you to practice regular physical activity?*	N = 204		N = 899		
	n	%	n	%	
Yes	71	34.8	304	33.8	0.099 *
No, the doctor hasn’t suggested it	103	50.5	496	55.2	
No, I haven’t spoken with any health worker	29	14.2	83	9.2	
I do not remember	1	0.5	16	1.8	
* advised you to keep a healthy weight or to lose weight?*	N = 209		N = 898		
	n	%	n	%	
Yes	77	36.8	216	24.1	<0.001 *
No, the doctor hasn’t suggested it	107	51.2	611	68.0	
No, I haven’t spoken with any health worker	25	12.0	68	7.6	
I do not remember	0	0	3	0.3	
* asked how much alcohol you drink? ^1^*	N = 99		N = 513		
	n	%	n	%	
Yes	23	23.2	75	14.6	0.035 *
No, nobody asked	60	60.6	377	73.5	
No, I haven’t spoken with any health worker	16	16.2	55	10.7	
I do not know	0	0	6	1.2	
** In the last 12 months, has a doctor ever advised you to quit smoking? ^2^**	N = 74		N = 188		
	n	%	n	%	
Yes, for health reasons	16	21.6	8	4.3	<0.001 *
Yes, for prevention	8	10.8	87	46.3	
Yes, for both reasons	8	10.8	18	9.6	
No	41	55.4	68	36.2	
I do not recall	1	1.4	7	3.7	
** Has a doctor ever**					
* measured your blood pressure?*	N = 210		N = 899		
	n	%	n	%	
Yes	195	92.8	857	95.3	0.120
No	14	6.7	33	3.7	
I do not recall	1	0.5	9	1.0	
* calculated your cardiovascular risk? ^3^*	N = 99		N = 349		
	n	%	n	%	
Yes	7	7.1	2	0.6	<0.001
No	85	85.8	319	91.4	
I do not recall	7	7.1	28	8.0	
** In the last 12 months, have you been vaccinated against the flu?**	N = 209		N = 899		
	n	%	n	%	
Yes	20	9.6	82	9.1	0.376 *
No	187	89.5	814	90.6	
I do not recall	2	0.9	3	0.3	

^1^ Question asked to respondents who stated they had drunk at least 1 alcohol unit in the previous month; ^2^ question asked to current smokers; ^3^ question asked to people aged 35–69, * Fisher’s exact test.

**Table 4:** Absolute and relative frequencies of responses to the PASSI questionnaire. Domain: Screening tests. Results are expressed as absolute values and percentages.

	Pap Smear					Mammogram				
	Bastogi		ASL Roma 1		*p*	Bastogi		ASL Roma 1		*p*
*Have you ever taken this test for preventive purposes at least once in life?*										
	N = 108		N = 400			N = 85		N = 332		
	n	%	n	%		n	%	n	%	
Yes	98	90.8	377	94.3	0.281 *	64	75.3	300	90.4	0.001 *
No	9	8.3	18	4.5		20	23.5	29	8.7	
I do not know	1	0.9	5	1.2		1	1.2	3	0.9	
*Have you ever received the letter from the local health unit inviting you to take this test?*										
	N = 100		N = 400			N = 62		N = 203		
	n	%	n	%		n	%	n	%	
Yes	67	67.0	189	47.2	<0.001	48	77.4	143	70.4	0.005
No	31	31.0	138	34.5		13	21.0	26	12.8	
I do not remember	2	2.0	73	18.3		1	1.6	34	16.8	
*Have you ever been advised by a doctor or healthcare professional to take the test regularly, in absence of symptoms?*										
	N = 104		N = 400			N = 69		N = 293		
	n	%	n	%		n	%	n	%	
Yes	58	55.8	291	72.8	<0.001 *	54	78.3	210	71.7	0.048
No	45	43.3	87	21.8		15	21.7	59	20.1	
I do not remember	0	0.0	19	4.7		0	0.0	24	8.2	
No, because already hysterectomized	1	0.9	3	0.7		-		-		
*Did you have to pay for the last test you got? ^1^*										
	N = 74		N = 343			N = 52		N = 252		
	n	%	n	%		n	%	n	%	
No	55	74.3	79	59.0	<0.001	43	82.7	67	26.6	<0.001
Just the ticket	8	10.8	69	20.1		5	9.6	77	30.6	
Yes	9	12.2	177	51.6		4	7.7	91	36.1	
I do not know	2	2.7	18	5.3		0	0.0	17	6.7	

^1^ Question asked to women who had taken the test within the previous 5 years (PAP test) and 2 years (mammogram), * Fisher’s exact test.

## Data Availability

Data may be obtained from a third party and are not publicly available. All data presented in the study are stored by the Local Health Unit Roma 1 and handled confidentially. Only the research team and related Institutions have access to the data.
